# Exposure to Plasma From Non-alcoholic Fatty Liver Disease Patients Affects Hepatocyte Viability, Generates Mitochondrial Dysfunction, and Modulates Pathways Involved in Fat Accumulation and Inflammation

**DOI:** 10.3389/fmed.2021.693997

**Published:** 2021-07-02

**Authors:** Elena Grossini, Divya Praveen Garhwal, Giuseppe Calamita, Raffaele Romito, Cristina Rigamonti, Rosalba Minisini, Carlo Smirne, Daniela Surico, Mattia Bellan, Mario Pirisi

**Affiliations:** ^1^Laboratory of Physiology, Department of Translational Medicine, University East Piedmont, Novara, Italy; ^2^AGING Project, Department of Translational Medicine, University East Piedmont, Novara, Italy; ^3^Laboratory of Cellular and Molecular Physiology and Pathophysiology, Department of Biosciences, Biotechnologies and Biopharmaceutics, University of Bari “Aldo Moro”, Bari, Italy; ^4^General Surgery Unit, Azienda Ospedaliera Maggiore della Carità University Hospital, Novara, Italy; ^5^Internal Medicine Unit, Department of Translational Medicine, University East Piedmont, Novara, Italy; ^6^Obstetrics and Gynecology Unit, Department of Translational Medicine, University East Piedmont, Novara, Italy

**Keywords:** biomarker, inflammasome, mitochondria, NAFLD, oxidative stress

## Abstract

Changes of lipidic storage, oxidative stress and mitochondrial dysfunction may be involved in the pathogenesis of non-alcoholic fatty liver disease (NAFLD). Although the knowledge of intracellular pathways has vastly expanded in recent years, the role and mechanisms of circulating triggering factor(s) are debated. Thus, we tested the hypothesis that factors circulating in the blood of NAFLD patients may influence processes underlying the disease. Huh7.5 cells/primary human hepatocytes were exposed to plasma from 12 NAFLD patients and 12 healthy subjects and specific assays were performed to examine viability, H_2_O_2_ and mitochondrial reactive oxygen species (ROS) release, mitochondrial membrane potential and triglycerides content. The involvement of NLRP3 inflammasome and of signaling related to peroxisome-proliferator-activating-ligand-receptor-γ (PPARγ), sterol-regulatory-element-binding-protein-1c (SREBP-1c), nuclear-factor-kappa-light-chain-enhancer of activated B cells (NF-kB), and NADPH oxidase 2 (NOX2) was evaluated by repeating the experiments in the presence of NLRP3 inflammasome blocker, MCC950, and through Western blot. The results obtained shown that plasma of NAFLD patients was able to reduce cell viability and mitochondrial membrane potential by about 48 and 24% (*p* < 0.05), and to increase H_2_O_2_, mitochondrial ROS, and triglycerides content by about 42, 19, and 16% (*p* < 0.05), respectively. An increased expression of SREBP-1c, PPARγ, NF-kB and NOX2 of about 51, 121, 63, and 46%, respectively, was observed (*p* < 0.05), as well. Those effects were reduced by the use of MCC950. Thus, in hepatocytes, exposure to plasma from NAFLD patients induces a NAFLD-like phenotype by interference with NLRP3-inflammasome pathways and the activation of intracellular signaling related to SREBP-1c, PPARγ, NF-kB and NOX2.

## Introduction

Non-alcoholic fatty liver disease (NAFLD) is currently the most common liver disease in the world and the second most common cause of liver transplantation in the United States (1, 2).

Our understanding of NAFLD pathogenesis and natural history has greatly expanded in the last decade, however, many issues remain unsolved. The liver plays a central role in regulating lipid homeostasis through processes that are strictly regulated by complex interactions between hormones, nuclear receptors, and transcription factors-related pathways. Their alteration may cause the retention of fat within the liver and the subsequent development of NAFLD (3, 4).

Another important player in the onset of NAFLD and its progression to non-alcoholic steatohepatitis (NASH) is mitochondrial dysfunction (5) that can alter the balance between prooxidants/antioxidants, leading to an increase of non-metabolized free fatty acids (FFAs) in the cytosol and the consequent induction of reactive oxygen species (ROS) production. A “primary” mitochondrial dysfunction has been proposed as one of the mechanisms of FFAs accumulation in the hepatocytes of NAFLD patients (6), however, an overload of FFAs into mitochondria may be able by itself to lead to dissipation of the membrane potential, loss of ATP synthesis capacity, and enhanced ROS generation (7). In addition, the accumulation of lipotoxic intermediates may promote inflammation and alter insulin signaling, facilitating the establishment of an insulin resistance state (5, 8).

Further topics of major interest regard the interference with pathways associated to recognition receptors including Toll-like receptors (TLRs) and NOD-like receptors (NLRs) (9), and downstream signaling involving peroxisome proliferator activating ligand receptors (PPAR), sterol regulatory element binding protein 1c (SREBP-1c), nuclear factor kappa-light-chain enhancer of activated B cells (NF-kB), and NADPH oxidase 2 (NOX2) (1, 10, 11).

In an “hormonocentric” view of NAFLD pathogenesis, all these processes may be triggered by circulating factors (12), that may represent diagnostic or prognostic biomarkers.

Based on these premises, we aimed to evaluate whether factors circulating in the blood of NAFLD patients may modulate one or more of the above-mentioned processes. Hence, in the present study we examined the effects of plasma of NAFLD patients on the viability and function of hepatocytes.

## Materials and Methods

### Patients and Controls

Experiments were conducted between October 2018 and March 2019 using plasma from 12 NAFLD patients and 12 healthy subjects. The use of excess plasma remaining from blood samples taken for the routinary clinical monitoring of NAFLD patients has been approved by the local Ethics Committee (Ethical Committee of the “Azienda Ospedaliera Maggiore della Carità” University Hospital in Novara). Patients and controls have given written informed consent for experimental use of pseudonymized clinical data and blood specimens. The work was carried out in accordance with The Code of Ethics of the World Medical Association (Declaration of Helsinki).

The clinical monitoring and plasma sampling of patients and controls were performed at Liver Clinic Unit, “Azienda Ospedaliera Maggiore della Carità” University Hospital in Novara.

### Culture of Huh7.5 Cells

The human hepatocellular carcinoma cell line Huh7.5 (Apath L.L.C New York, USA) (13) was maintained in Dulbecco's modified Eagle's medium (DMEM; Sigma, Milan, Italy) supplemented with 10% fetal bovine plasma (FBS; Euroclone, Pero, Milan, Italy), 2 mM L-glutamine (Euroclone), 1% penicillin-streptomycin (P/S; Euroclone), at 37°C with 5% CO_2_ in incubator.

### Isolation of Hepatocytes From Human Liver Biopsy Specimens

From October 2018 until November 2019, 10 liver samples (i.e., 50–100 g each) were obtained from patients undergoing surgery at General Surgery Unit, Azienda Ospedaliera Maggiore della Carità University Hospital, in Novara, due to primary or metastatic liver resectable tumor.

The specimens were acquired from the non-neoplastic portion of the liver parenchyma resected during the surgery. All tissue donors gave written informed consent for experimental use of pseudonymized clinical data and liver specimen prior to surgery. Then fresh samples were transferred on ice cold physiologic saline solution to Physiology laboratory, and hepatocytes were immediately isolated, as previously performed (14, 15). Briefly, liver specimens were minced with a scalpel and undergone a two steps collagenase procedure; the cell pellet was, then, centrifugated at 300 g for 5 min and the cell pellet was filtered through a nylon mesh. Thereafter, hepatocytes were pooled and seeded into plates coated with collagen-I (Sigma), and maintained in DMEM/HAM'S F-12 (Euroclone) supplemented with 10% FBS (Euroclone), 100 U/ml penicillin (Sigma), 0.1 mg/ml streptomycin (Sigma), and 2 mM L-glutamine (Sigma). Finally, they were transferred into 75-cm^2^ culture flasks (Euroclone) in incubator under standard conditions. The culturing was performed until passage 15 (16).

### Experimental Protocol

To evaluate the effects of plasma samples taken from NAFLD patients and healthy subjects, on cell viability (MTT Assay), mitochondrial membrane potential (JC-1 Assay), H_2_O_2_ release (ROS-Glo H_2_O_2_,) mitochondrial ROS (mitoROS) release, triglycerides content (Triglyceride assay) and protein expression (Western Blot) in Huh7.5 cells/primary human hepatocytes, co-culture experiments were performed, by using specific Transwell inserts (Supplementary Figure 1A).

For the experiments, plasma samples from 12 NAFLD patients and 12 healthy subjects were plated in the apical compartment of the insert and left to act for 3 h, while, Huh7.5 cells/primary human hepatocytes were plated in the basal compartment. The 3 h time limit for stimulation was chosen based on preliminary time-course experiments during which Huh7.5 cells were exposed to plasma for 3, 12, and 24 h, showing that cell viability was drastically reduced already at 12 h. In addition, preliminary experiments were performed on both Huh7.5 cells and primary human hepatocytes with 5, 10, and 20% plasma calculated in relation to the total volume of each insert. In those experiments the effects of plasma from NAFLD patients and healthy subjects on cell viability and H_2_O_2_ release were measured (Supplementary Figure 1B). The results obtained allowed us to select the proper plasma concentration to be used for all next experiments performed on Huh7.5 cells. Furthermore, in some experiments, 1 nM inflammasome NLRP3 inhibitor (MCC950) was administrated to Huh7.5 cells for 30 min alone or before 200 pM TNFα (for 3 h) in co-stimulation with plasma. Some samples of Huh7.5 cells and primary human hepatocytes were not treated with plasma and were used as “control.” At the end of stimulations, various assays were performed (Supplementary Figure 1A). All experiments were conducted in triplicate and repeated at least five times.

### 1) Cell Viability

Cell viability was examined in Huh7.5 cells and primary human hepatocytes by using the 1% 3-[4,5-dimethylthiazol-2-yl]-2,5-diphenyl tetrazolium bromide (MTT; Life Technologies Italia, Monza, Italy; catalog number CT02) dye, as previously performed (17–21) and described in Supplementary Material. Cell viability was determined by measuring the absorbance through a spectrometer (VICTOR™ X Multilabel Plate Reader; PerkinElmer) with a wavelength of 570 nm and cell viability was calculated by setting control cells as 100%.

### Mitochondrial Membrane Potential Measurement

Mitochondrial membrane potential measurement in Huh7.5 cells was performed with JC-1 assay (17, 19–22). The detailed description of methods is reported in Supplementary Material. The mitochondrial membrane potential was determined by measuring the red (excitation 550 nm/emission 600 nm) and green (excitation 485 nm/emission 535 nm) fluorescence through a spectrometer (VICTOR™ X Multilabel Plate Reader; PerkinElmer). The data were normalized vs. control cells.

### 2) Mitochondrial ROS Quantification

MitoROS production was determined through the Cayman's Mitochondrial ROS Detection Assay Kit (Cayman Chemical; catalog number 701600), as previously performed (23) and described in Supplementary Material. The MitoROS production was measured with an excitation and emission wavelength of 480 and 560 nm, respectively, by using a spectrophotometer (VICTOR™ X Multilabel Plate Reader; PerkinElmer). The data were normalized vs. control cells.

### 3) ROS-Glo H_2_O_2_ Quantification

H_2_O_2_ production was determined by the ROS-Glo H_2_O_2_ Assay, following the manufacturer's instructions (Promega Corporation; Padova, Italy; catalog number G8820) (24, 25). The detailed description of methods is reported in Supplementary Material. The H_2_O_2_ production was quantified as relative luminescence by using a spectrophotometer (VICTOR™ X Multilabel Plate Reader; PerkinElmer). The data were normalized vs. control cells.

### 4) Triglycerides Quantification

Triglycerides measurement was performed with a specific kit (Cayman Chemical; catalog number 10010303) and as described in Supplementary Material. The triglycerides content was detected following the manufacturer's instructions through a spectrometer (VICTOR™ X Multilabel Plate Reader; PerkinElmer) at excitation/emission wavelengths of 530–550 nm (22). The value of each sample was quantified in respect to triglycerides standard curve and expressed as triglycerides content (mg/dl).

### 5) Cell Lysates

For protein expression/activation, Huh7.5 cells were stimulated as described for various assays. For the experiments, 400000 Huh7.5 cells/insert in 6-Transwell plate were plated. At the end of stimulation, Huh7.5 cells were lysed in iced Ripa buffer supplemented with sodium orthovanadate (2 mM; Sigma) and protease inhibitors cocktail (1 mM; Thermo Fisher Scientific) and phenylmethanesulfonyl fluoride (1 mM; Sigma). The extracted proteins were quantified through bicinchoninic acid protein (BCA, Pierce) and used for electrophoresis and immunoblotting studies (16–22). Due to shortage of plasma specimens, experiments were conducted with plasma taken from three NAFLD patients and three healthy subjects and were repeated at least three times.

### Western Blot Analysis

Cell lysates (30 μg protein each sample) were dissolved in 5 × Laemmli buffer, boiled for 5 min and resolved in 10% sodium dodecyl sulfate polyacrylamide gel electrophoresis gels (Bio-Rad Laboratories). After electrophoresis they were transferred to polyvinylidene fluoride membranes (Bio-Rad Laboratories) and incubated overnight at 4°C with specific primary antibodies: anti Nf-kB (p-50; 1:1,000; Santa Cruz Biotechnology, catalog number sc-8414), anti gp91-phox (NOX2; 1:1,000; Santa Cruz Biotechnology, catalog number sc-130543), anti PPAR-γ (1:1,000; Santa Cruz Biotechnology, catalog number sc-271392), anti SREBP-1c (1:1,000; Santa Cruz Biotechnology, catalog number sc-13551). The membranes were washed and then incubated with horseradish peroxidase-coupled goat anti-rabbit IgG (Sigma), peroxidase-coupled rabbit anti-goat IgG and horseradish peroxidase-coupled goat anti-mouse IgG (Sigma) for 45 min and were developed through a non-radioactive method using Western Lightning Chemiluminescence (PerkinElmer Life and Analytical Sciences). Phosphorylated protein expression was calculated as a ratio toward β-actin (1:5,000; Santa Cruz Biotechnology; catalog number sc-47778) detection.

### Statistical Analysis

Statistical analysis was performed using STATVIEW version 5.0.1 for Microsoft Windows (SAS Institute Inc., Cary NC, USA). Data were checked for normality before statistical analysis. All data are presented as means ± standard deviation (SD) of five independent experiments for each experimental protocol. Differences between groups were analyzed by one-way ANOVA and Bonferroni *post hoc* tests. The threshold for statistical significance was 0.05 (two-tails).

## Results

NAFLD patients were seven males and five females, aged 51 ± 14 years, had a body mass index (BMI) of 31.1 ± 4.0 kg/m^2^, plasma alanine aminotransferase (ALT) concentration of 43 ± 29 U/L, plasma total cholesterol of 182 ± 30.4 mg/dl, liver stiffness and a controlled attenuation parameter measured by FibroScan® of 7.1 ± 1.9 kPa and 297 ± 45 dB/m, respectively. Healthy subjects were seven males and five females, aged 24.2 ± 0.4 year, had a BMI 22.9 ± 2.9 kg/m^2^, plasma ALT concentration of 23 ± 4 U/L, plasma total cholesterol of 167 ± 19 mg/dl, liver stiffness and a controlled attenuation parameter measured by FibroScan® of 5.4 ± 1 kPa and 186 ± 27 dB/m, respectively. Significant differences were observed in age, BMI, ALT, controlled attenuation parameter and liver stiffness measured between NAFLD and non-NAFLD patients (*p* < 0.05).

**Figure 1:**
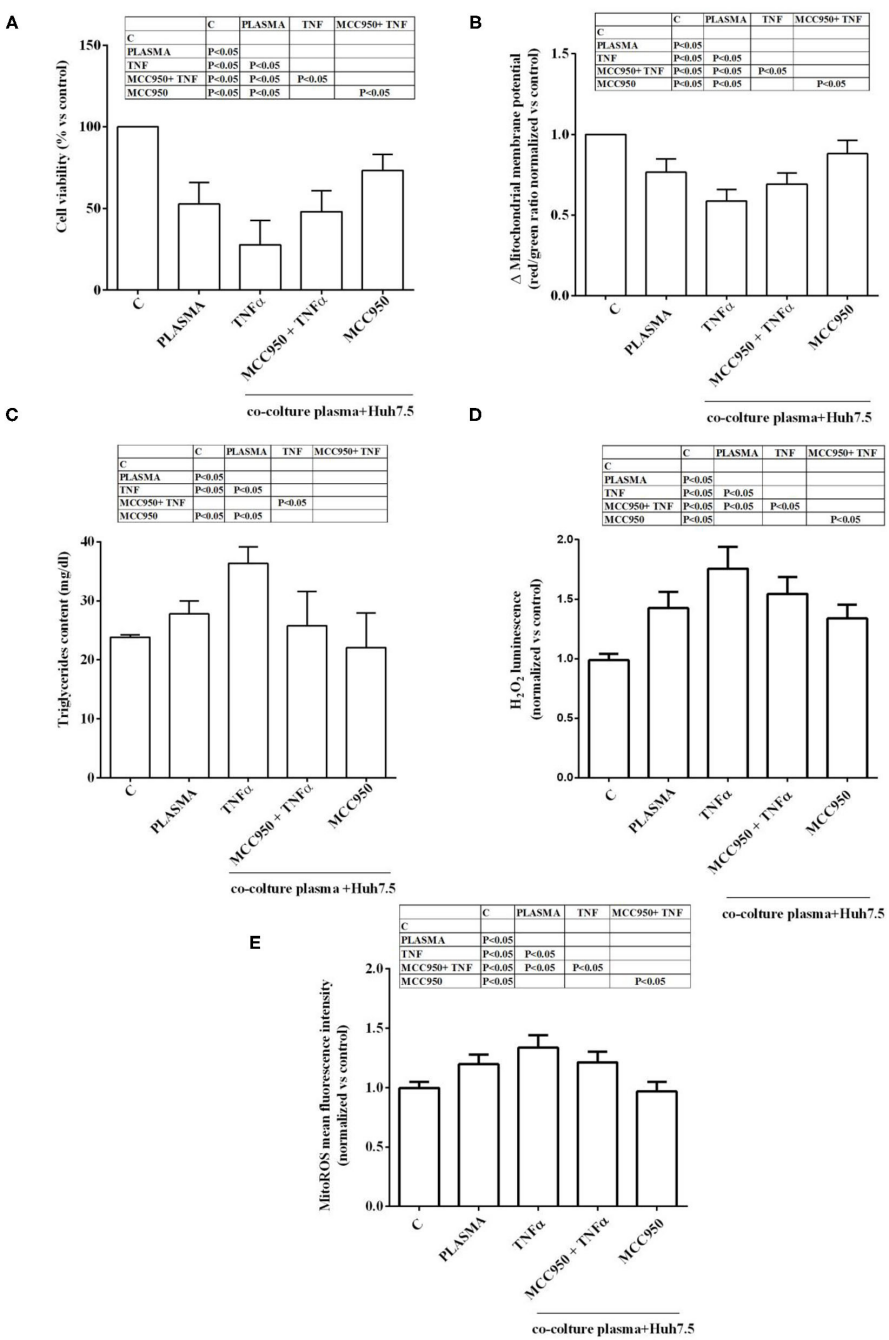
Effects of NAFLD plasma on Huh7.5 cell viability **(A)**, mitochondrial membrane potential **(B)**, triglycerides content **(C)**, H_2_O_2_ release **(D)** and mitochondrial ROS (mitoROS, **E**). C=non-treated cells. MCC950 (NLRP3 inflammasome inhibitor, 1 nM for 30 min); TNFα (200 pM for 3 h). Reported data are means ± SD of five independent experiments for each experimental protocol.

A dose response study was executed in order to choose the proper plasma concentration for the *in vitro* experiments. As shown in Supplementary Figure 2, plasma from NAFLD patients was able to reduce cell viability and increase H_2_O_2_ release in both Huh7.5 cells and primary human hepatocytes. In addition, a grading response was seen by using 5–20% plasma. Indeed, as regarding primary human hepatocytes viability, it amounted to 25, 34, and 43% about, in cells treated with 20, 10, and 5% NAFLD plasma, respectively. At the same time ROS release increased nearly by 100, 76, and by 56%. Moving on Huh7.5 cells, cell viability amounted to 34, 43, and 53% about, in cells treated with 20, 10, and 5% NAFLD plasma, respectively, whereas ROS release increased nearly by 80, 60, and 40%. Although the highest effects were observed after the treatment with the 20% plasma concentration, we decided to choose the 5% plasma concentration for all subsequent experiments in order to save plasma. As described in Figure 1, in Huh7.5 cells, plasma of NAFLD patients was able not only to reduce the cell viability but also mitochondrial membrane potential, by 47.25 ± 13.2% and 23.5 ± 0.8%, respectively. The finding of a reduction of mitochondrial membrane polarization would confirm the harmful effects elicited by NAFLD plasma. It is to note that those effects were accompanied by an increase not only of H_2_O_2_ release amounting to 45 ± 1.3%, but also of mitoROS release of 20.2 ± 0.7%. In addition, an increase of triglycerides content of 27.8 ± 2.1% was observed, as well.

In the presence of MCC950, the NLRP3 inflammasome inhibitor, the effects of NAFLD plasma on Huh7.5 cells were counteracted. Hence, cell viability and mitochondrial membrane potential were increased in comparison with findings obtained in Huh7.5 cells treated with NAFLD plasma alone (Figures 1A,B). Moreover, ROS and mitoROS release were reduced, as well as, the triglycerides content (Figures 1C–E).

**Figure 2:**
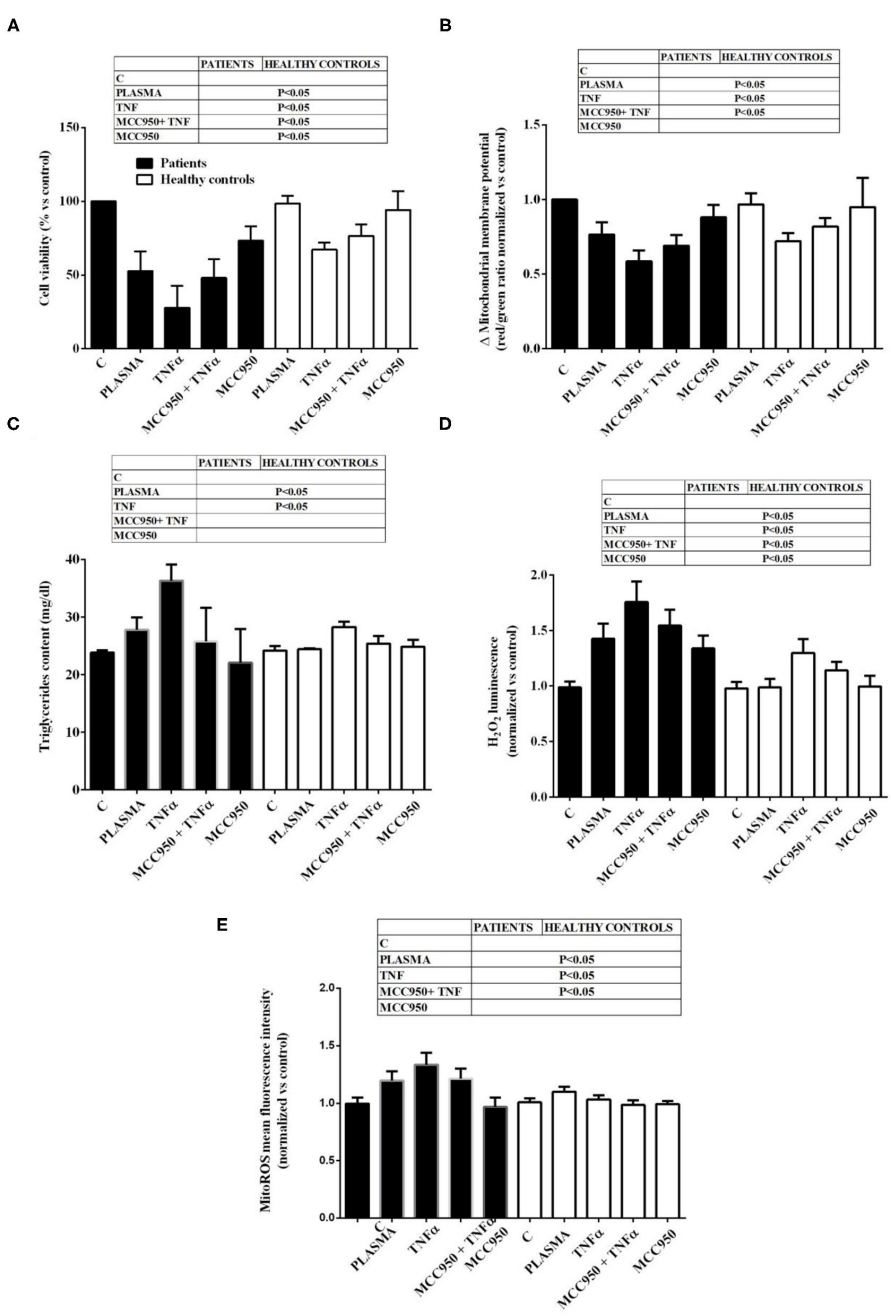
Comparison between the effects of NAFLD plasma and of plasma from healthy subjects on Huh7.5 cell viability **(A)**, mitochondrial membrane potential **(B)**, triglycerides content **(C)**, H_2_O_2_ release **(D)** and mitochondrial ROS (mitoROS, **E**). Abbreviations are as described in Figure 1. Reported data are means ± SD of five independent experiments for each experimental protocol.

It is to note that all the effects of NAFLD plasma on Huh7.5 cells were higher than those caused by plasma from healthy subjects (Figure 2). Also, TNFα was able to reduce cell viability, mitochondrial membrane potential, to increase triglycerides content and promote hydrogen peroxide and mitoROS release in a stronger way in the presence of NAFLD plasma than healthy subjects' plasma. The NLRP3 inflammasome inhibitor, MCC950, was so effective in preventing the deleterious effects of NAFLD plasma that no differences could be observed between NAFLD plasma and healthy subjects' plasma, as regarding mitochondrial membrane potential, triglycerides content and mitoROS release.

**Figure 3:**
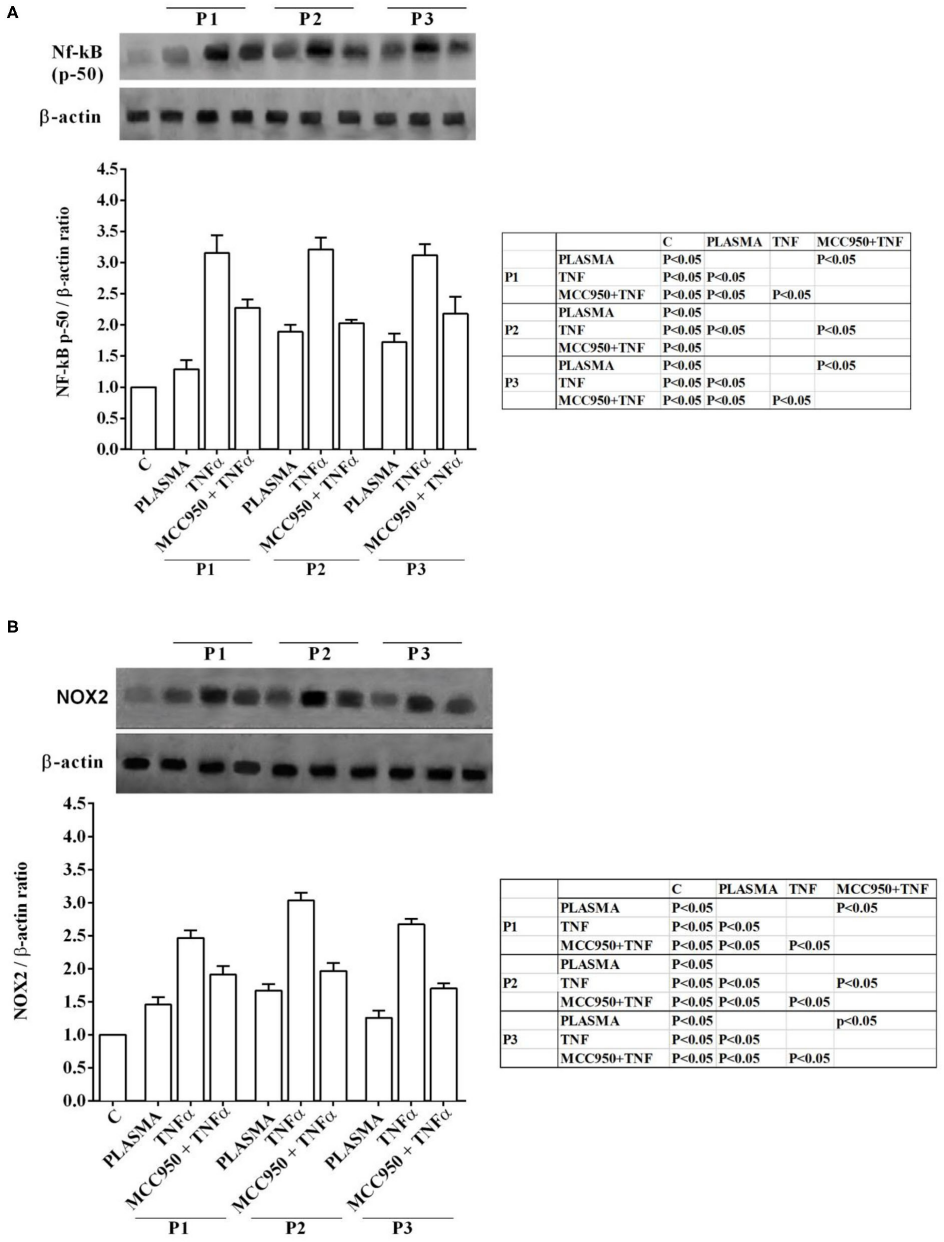
Effects of NAFLD plasma on NF-kB **(A)** and NOX2 **(B)** expression in Huh7.5 cells. P, patient; M, MCC950 (1 nM, for 30 min). Other abbreviations are as described in previous Figures. In the densitometric analysis, all values are normalized vs. control which is normalized, as well, and considered as 1. They are shown as fold changes vs. control. Reported data are means ± SD of five independent experiments for each experimental protocol.

**Figure 4:**
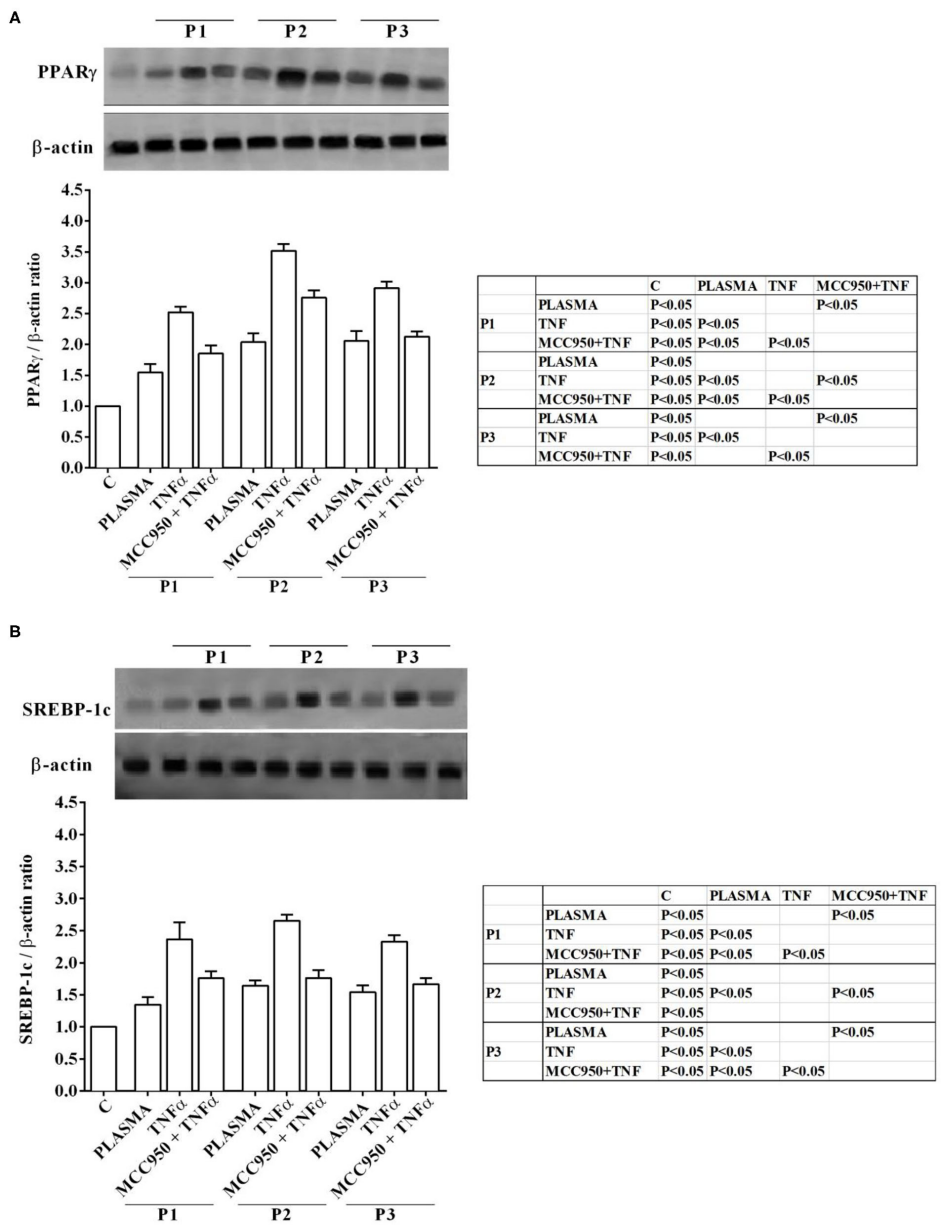
Effects of NAFLD plasma on PPARγ **(A)** and SREBP-1c **(B)** expression in Huh7.5 cells. P, patient; M, MCC950 (1 nM, for 30 min). Other abbreviations are as described in previous Figures. In the densitometric analysis, all values are normalized vs. control which is normalized, as well, and considered as 1. They are shown as fold changes vs. control. Reported data are means ± SD of five independent experiments for each experimental protocol.

Western blot analysis performed on Huh7.5 cells treated with NAFLD plasma at 5% concentration showed the involvement inflammatory pathways and of an intracellular signaling related to lipid accumulation in liver during NAFLD. Indeed, in Huh7.5 cells treated with plasma of NAFLD patients, an increase in the expression of NF-kB, NOX2, PPARγ and SREBP-1c of 28.7 ±14.8%, 31,8 ± 16,1%, 55 ± 13%, 34,7 ± 13%, respectively, was observed (Figures 3, 4).

In our experiments we used TNFα as positive “inflammatory” control to stimulate Huh7.5 cells treated with plasma of both NAFLD patients and healthy subjects. As observed in previous experiments, also in Western blot analysis all effects of plasma of NAFLD patients were potentiated by TNFα and reduced by MCC950 (Figures 1–4).

In addition, all the above effects observed in Huh7.5 cells treated with plasma of NAFLD patients were accompanied by an increase in the expression of NF-kB, NOX2, PPARγ and SREBP-1c of 28.7 ±14.8%, 31,8 ± 16,1%, 55 ± 13%, 34,7 ± 13%, respectively (Figures 3, 4). All effects of plasma of NAFLD patients were potentiated by TNFα and reduced by MCC950 (Figures 1–4).

## Discussion

The present paper documents that exposure of hepatocytes to plasma from NAFLD patients was able to affect cell viability and to modulate intracellular pathways *in vitro* in the direction predicted by current hypotheses on NAFLD pathogenesis.

In recent years, several *in vitro* model of NAFLD of increasing complexity have been proposed, from culture of a single cell type to 3D cocultures and body-on-a-chip systems (26). Human hepatoma cell lines are easy to maintain and may be apt for multiparametric evaluation of steatosis (27), though their enzymatic profile may differ significantly from that of normal hepatocytes. On these premises, we decided to set a system in which human hepatoma cells could be maintained at ease varying conditions in which they were grown by using specific stimulant and inhibitor molecules. It should be of particular interest and represent a touch of originality the use of plasma for treating hepatocytes, Hence, almost all previous studies have been performed by simulating an *in vitro* condition of NAFLD milieu, using mixed FFAs solutions. Here, we aimed to examining the role of any circulating factors playing a pathophysiological role in the onset of NAFLD. The Huh7.5 cell line that we employed in our experiments has gained popularity for being highly permissive for hepatitis C virus replication *in vitro* (28). Moreover, some experiments have been performed in human primary hepatocytes, as well, to set up the optimal experimental conditions and to confirm the results obtained in Huh7.5 cells.

It is to note that the method employed for hepatocytes isolation is the one that was reported to result in hepatocytes populations of high purity with retained physiological activity *in vitro* (14).

In both Huh7.5 cells and human primary hepatocytes we tested 5, 10, and 20% NAFLD and non-NAFLD plasma samples on cell viability and H_2_O_2_ release, so that we could select the proper plasma concentration to be used in all other experiments performed on Huh7.5 cells only. To culture medium of Huh7.5 cells we also added TNFα, to reproduce *in vitro* conditions mimicking the steatohepatitis milieu (29), where TNFα plays a major role (30).

Furthermore, experiments were conducted in the presence or absence of the potent and selective inhibitor MCC950, which targets directly the NLRP3 ATP-hydrolysis motif for inflammasome inhibition (31).

Regarding the loss of cell viability that we observed after exposure to NAFLD plasma, it was presumably due to the differences in triglycerides content and oxidants release observed in Huh7.5 cells. It is to note that H_2_O_2_ and mitoROS have widely been considered as main contributors to liver injury and disease progression in NAFLD (32–35).

These effects were amplified in the presence of TNFα and reduced by the pretreatment with MCC950. It is noteworthy that in similar *in vitro* models, TNFα appears to mediate an increase of mitochondrial Ca^2+^ leading to production of ATP and ROS, with the shedding of TNF receptor 1 (which acts as a decoy) limiting the propagation of the inflammatory response (36). Our results would indicate the potentiation by TNFα of the harmful effects elicited by not yet clarified “inflammatory” factors circulating in the plasma of NAFLD patients, a mechanism that was counteracted by the NLRP3 inflammasome inhibition.

Following the “multiple-hit” hypothesis, hepatic lipid overload would induce the overproduction of oxidants that could be detrimental for DNA, lipids and proteins and lead to the accumulation of the so called “damage-associated molecular pattern” (DAMPS), which would induce liver injury. In addition, the impairment of the electrons transfer chain and the fall of mitochondrial membrane potential could be followed by cell death (35).

Mitochondrial membrane potential is essential for cells, enabling them to store energy: a long-lasting drop of mitochondrial potential is deleterious to cell viability, possibly more because of interference by products of ATP hydrolysis than by ATP in itself. About this issue it is to note that the increased mitoROS release we have observed was accompanied by the fall of mitochondrial membrane potential in Huh7.5 cells.

Interestingly, we also found an increase of SREBP-1c and PPARγ expression, which are involved in the intracellular triglycerides accumulation (1) and an increase of NF-kB and NOX2. As concerning SREBP-1c, it has been found to be involved in mitochondrial oxidants release through AQP8 upregulation (36). NF-kB is responsible for the transcription of pro-inflammatory molecules; among other stimuli like SREBP-1c (37). Also, NOX enzymes, especially NOX1, NOX2, and NOX4, have been associated with liver injuries in NAFLD both *in vivo and in vitro*, through the increased ROS release and the reciprocal interaction between different NOX enzymes and mitochondria (38).

The data we have obtained with MCC950 highlight the involvement of NLRP3 inflammasome in eliciting the harmful effects of NAFLD plasma in Huh7.5 cells. Our findings are in agreement with previous observations about the role of NOD-like receptors in NAFLD pathogenesis. In fact, the release by dying hepatocytes of DAMPS can be detected by members of NOD-like receptors, such as the NLRP3 inflammasome. Its activation is followed by cleavage of pro-caspase-1 into active caspase-1, which, in turn, cleaves pro-IL-1β into mature IL-1β (39, 40). Overall, the activation of NLRP3 inflammasome may potentiate a proinflammatory condition, resulting into stellate cells activation and transformation in a myofibroblastic phenotype. The fact however, that the only some effects of NAFLD plasma were abolished by MCC950, whereas others were just reduced, would suggest, on the one side, the important role played by NLRP3 inflammasome, and on the other side, the existence of other mechanisms which could trigger the damage. As also previously reported and confirmed by our results, intracellular pathways related to SREBP-1c and PPARγ could be involved. In addition, the modulation of inflammatory/anti-inflammatory cytokines, like interleukin 6 or adiponectin, could play a role and could be object of future studies.

Interestingly, the present data suggest the existence of an association between the activation of the NLRP3 inflammasome and that of intracellular pathways associated with SREBP-1c, PPARγ, NOX2 and NF-kB. In the presence of the inhibitor MCC950, the expression of all the above proteins was reduced in Huh7.5 cells exposed to NAFLD plasma. Data about SREBP-1c are also in agreement with previous findings in HepRG cells (41). Thus, it could be hypothesized a role for the NLRP3 inflammasome in the regulation of triglycerides accumulation via involvement of SREBP-1c and PPARγ, and in the onset of oxidative stress. Concomitantly or alternatively, the NLRP3 inflammasome may be responsible for oxidants release through signaling involving NOX2 and NF-kB. Future studies could be organized aimed at the analysis of other intracellular pathways related to all the above pathways and their relationships. In this context, it would be of interest to examine the role of AMP-activated protein kinase (AMPK), which is crucial for the regulation of fat mebabolism in liver.

One limitation of the present study is that NAFLD patients and healthy subjects were different as regarding BMI and age. This bias is somewhat inevitable, since NAFLD and NAFLD progression are strongly related to BMI and age. Moreover, sample size could be increased. The hepatoma cell line we used is not fully comparable to those of normal hepatocytes, though preliminary experiments performed on primary human hepatocytes (data not shown) confirmed the results obtained regarding cell viability and oxidant release. One may also argue that the model does not allow the identification of any specific pathogenic factor(s) and/or of putative biomarker(s); on the other hand, it has the advantage of being free of bias on the kind of substances involved. As said, our aim was to examine the role of any circulating factor possibly involved in the onset of NAFLD without venturing in the attempt of identifying all possibleculprits. Indeed, this is the first study performed by using NAFLD plasma on Huh7.5 cells and aimed at the analysis of cell viability, mitochondrial function and oxidants release. In addition, the role of main pathways involved in NAFLD onset has been investigated by performing experiments in the presence of an inhibitor and through Western Blot. The findings of harmful effects elicited by NAFLD plasma on hepatocytes would represent the starting point for the execution of future studies aimed at deepening the aforementioned issues. In particular, the application of proteomic and metabolomic profiling technologies to NAFLD plasma might contribute to identify any circulating factor(s) (microRNA, extracellular vesicles), whereas, *in vitro* experiments could be organized to increase the knowledge about the intracellular mechanisms (SREBP-1c, PPARγ, inflammasome molecules, AMPK).

## Conclusions

In summary, we have established an *in vitro* model in which exposure to NAFLD plasma, in agreement with what predicted by “hormonocentric” theories on NAFLD pathogenesis (12, 42) resulted in loss of cell viability and activation of pathways known to be involved in inflammation and tissue damage. Substances present in NAFLD plasma could have affected lipid metabolism of Huh7.5 cells: triglycerides accumulation activated as downstream signaling involving SREBP-1c and PPARγ may have acted as a starting point. Hence, accumulation of triglycerides may have hampered mitochondrial function either directly or through increased oxidants release, NOX2 and NLRP3 inflammasome-related pathways.

## Data Availability Statement

The original contributions presented in the study are included in the article/Supplementary Material, further inquiries can be directed to the corresponding author/s.

## Ethics Statement

The studies involving human participants were reviewed and approved by Ethical Committee of the Azienda Ospedaliera Maggiore della Carità University Hospital in Novara. The patients/participants provided their written informed consent to participate in this study.

## Author Contributions

EG and MP: concenptualization and funding acquisition. DG, RM, RR, CR, CS, and MB: methodology and investigation. DS, MB, and MP: resources. DG, GC, CR, CS, and MB: formal analysis. EG, DG, GC, RR, CR, RM, CS, DS, MB, and MP: supervision, validation, visualization, writing/review, and editing. EG, GC, and MP: writing/original draft preparation. All authors involved in editing the paper and had final approval of the submitted and published versions.

## Conflict of Interest

The authors declare that the research was conducted in the absence of any commercial or financial relationships that could be construed as a potential conflict of interest.
