# Supplementary material for: Exposure to Plasma From Non-alcoholic Fatty Liver Disease Patients Affects Hepatocyte Viability, Generates Mitochondrial Dysfunction, and Modulates Pathways Involved in Fat Accumulation and Inflammation
Source: Front Med (Lausanne). 2021 Jul 2;8:693997. doi: 10.3389/fmed.2021.693997 (PMC8282995; doi:10.3389/fmed.2021.693997)
Supplement: Supplementary file 1 [file Data_Sheet_1.docx]

**Supplementary Materials**

**Cell viability**

Cell viability was examined in Huh7.5 cells and primary human hepatocytes by using the 1% 3-[4,5-dimethylthiazol-2-yl]-2,5-diphenyl tetrazolium bromide (MTT; Life Technologies Italia, Monza, Italy; catalogue number CT02) dye. For the experiments, 200000 Huh7.5 cells or primary human hepatocytes/well were plated in 24-Transwells plates in complete medium. Huh7.5 cells and primary human hepatocytes were treated as described in the Experimental rotocol. After each treatment, the medium was removed, and fresh culture medium without red phenol and FBS and with MTT dye was added to the 96-well plates containing the cells and incubated for 2 h at 37°C in an incubator. Thereafter, the medium was removed, and an MTT solubilization solution (dimethyl sulfoxide; DMSO; Sigma) in equal volume to the original culture medium was added and mixed in a gyratory shaker until the complete dissolution of formazan crystals.

**Mitochondrial ROS (MitoROS) quantification**

MitoROS production was determined through the Cayman’s Mitochondrial ROS Detection Assay Kit (Cayman Chemical; catalogue number 701600). For the experiments, 200000 Huh7.5 cells/well were plated in 24-Transwells plates in complete medium, and then the same experimental protocol followed for MTT, JC-1 and ROS methods was used. Briefly, after treatments, the reactions were stopped by removing the culture media and addition of 120 µl of Cell Based Assay Buffer. After that, the Buffer was aspirated and 100 µl of Mitochondrial ROS Detection Reagent Staining Solution, was added in each well and incubated at 37°C, protected from light for 20 min. After this time, the Staining Solution was removed and each well was washed with 120 µl of PBS for three times.

**ROS-Glo H_2_O_2_ quantification**

The H_2_O_2_ production was determined by the ROS-Glo H_2_O_2_ Assay, following the manufacturer’s instructions (Promega Corporation; Padova, Italy; catalogue number G8820). H_2_O_2_ is convenient to quantify because among various ROS it has the longest half-life in cultured cells. For the experiments, 200000 Huh7.5 cells or primary human hepatocytes/well were plated in 24-Transwells plates in complete medium, and then the same experimental protocol followed for MTT, JC-1, and mitoROS assays was used. However, for this protocol, each plasma sample was incubated with 25 µM H_2_O_2_ Substrate Solution and left in incubator for 3 h. At the end of each stimulation, 100 µl of ROS-Glo Detection Solution, was added in each well and incubated for 20 min at room temperature. During this period, the ROS-Glo Detection Solution, containing Ultra-Glo Recombinant Luciferase and _D_-Cysteine, is converted to luciferin by the _D_-Cysteine, producing luciferin. The produced luciferin reacts with Ultra-Glo Recombinant Luciferase to generate a luminescent signal that is proportional to H_2_O_2_ concentration.

**Triglycerides quantification**

Triglycerides measurement was performed with a specific kit (Cayman Chemical; catalogue number 10010303). For the experiments, 400000 Huh7.5 cells/insert in 6-Transwell plate were plated and treated, as described for previous assays. Briefly, after treatments, cells were lysed by using the Standard Diluent 1X and a rubber policeman and centrifuged at 10000 g for 10 min at 4°C. After centrifugation, the supernatant was collected and further diluted 1:2 with Standard Diluent. Ten μl of sample was transferred to a 96‐well plate and 150 µl of diluted Enzyme Mixture solution was added in order to start the reaction. After 15 min of incubation at room temperature, the triglycerides content was detected following the manufacturer's instructions through a spectrometer (VICTOR™ X Multilabel Plate Reader; PerkinElmer) at excitation/emission wavelengths of 530–550 nm. The value of each sample was quantified in respect to triglycerides standard curve and expressed as triglycerides content (mg/dl). Experiments were conducted in triplicate and repeated at least five times.

Supplementary Figures 1


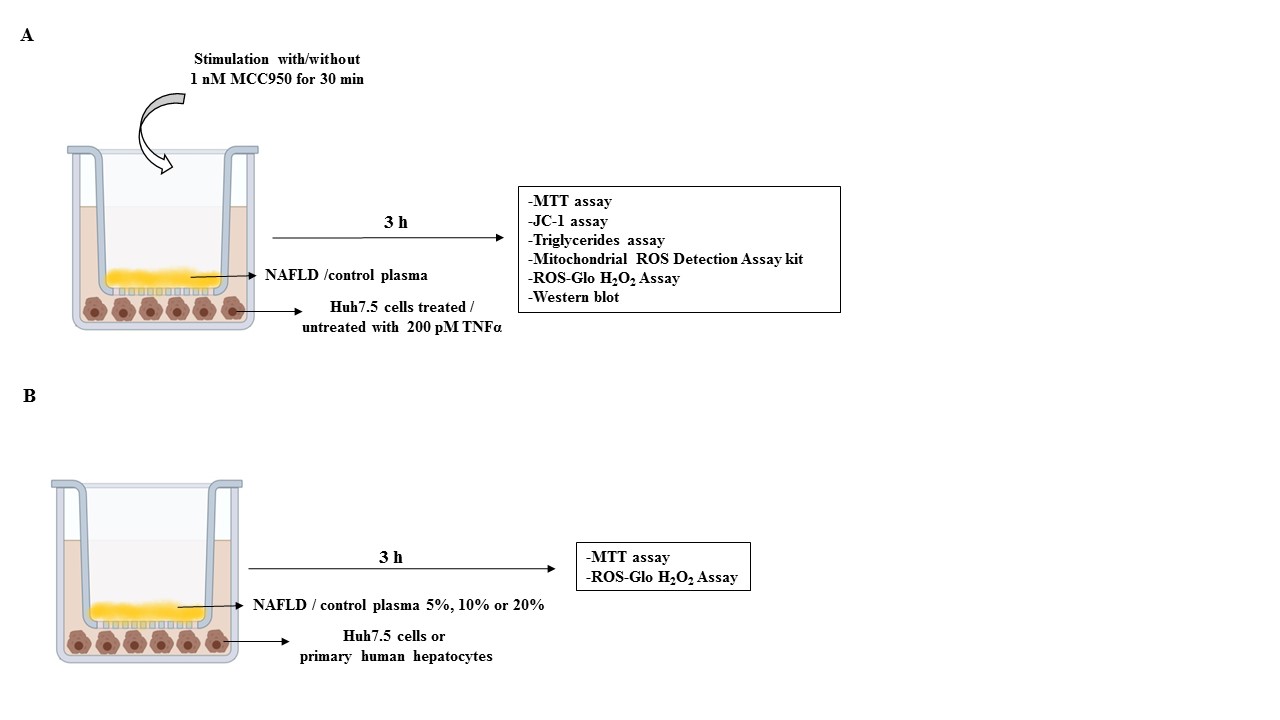


**Supplementary Figure 1.** Flowchart of experimental protocol. In **A**, effects of plasma on Huh7.5 cells. In **B**, effects of plasma on Huh7.5 cells and primary human hepatocytes. NAFLD: non-alcoholic fatty liver disease; JC-1: 5,51,6,61-tetrachloro-1,11,3,31 tetraethylbenzimidazolyl carbocyanine iodide; MCC950: NLRP3 inflammasome inhibitor; MTT: 3-[4,5-dimethylthiazol-2-yl]-2,5-diphenyl tetrazolium bromide; ROS: reactive oxygen species; TNF-α: tumor necrosis factor α.

Supplementary Figures 2


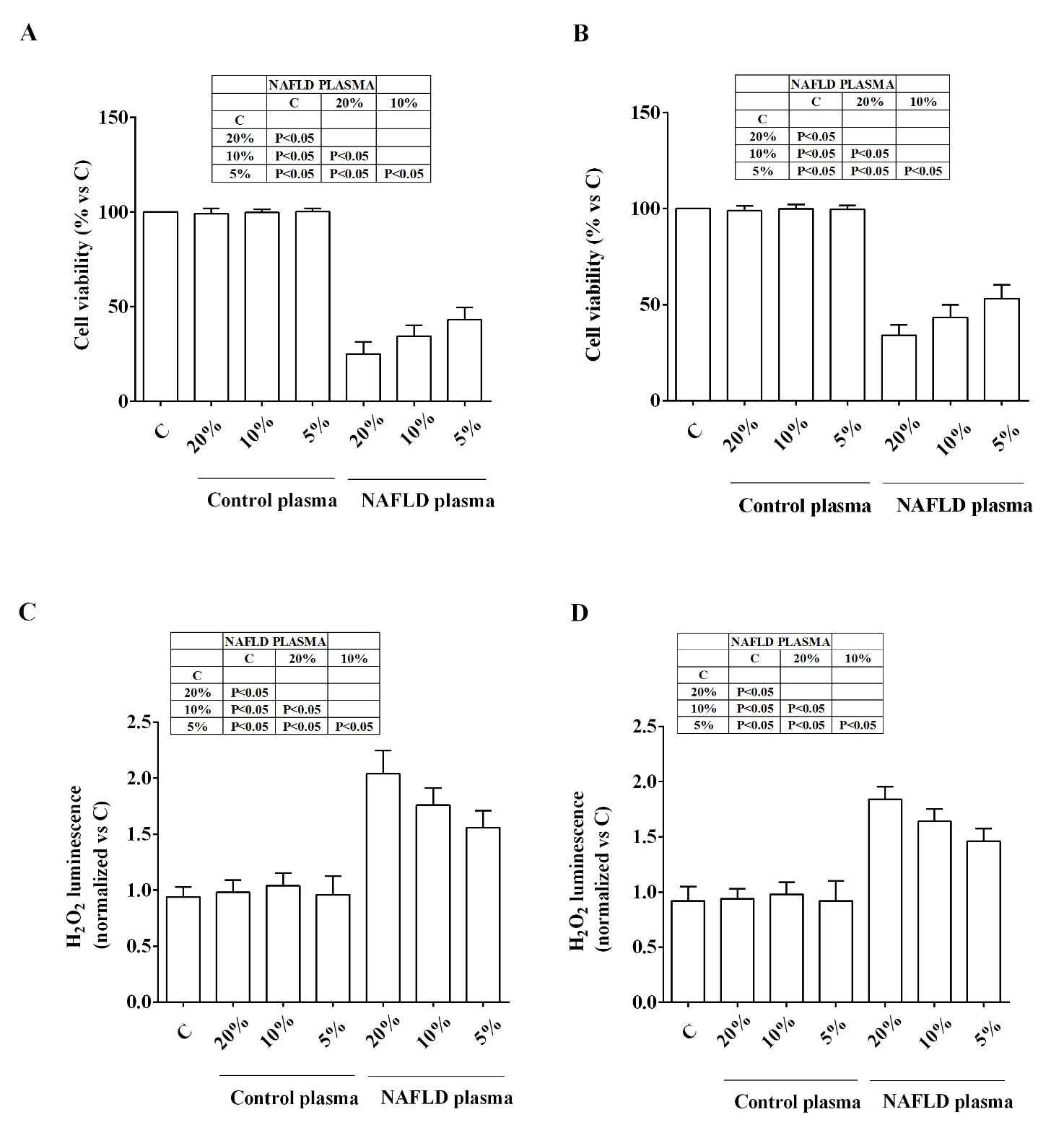


**Supplementary Figure 2**. Effects of plasma from NAFLD patients and healthy subjects (control plama) at different concentrations on cell viability (**A, B**) and H_2_O_2_ release (**C, D**) in primary human hepatocytes (**A, C**) and Huh7.5 cells (**B, D**). C= non-treated cells. Reported data are means ± SD of five independent experiments for each experimental protocol.
